# Plasmonic nanosensor based on multiple independently tunable Fano resonances

**DOI:** 10.3762/bjnano.10.243

**Published:** 2019-12-17

**Authors:** Lin Cheng, Zelong Wang, Xiaodong He, Pengfei Cao

**Affiliations:** 1 Institute of Optoelectronics & Electromagnetic Information, Lanzhou University, Lanzhou 730000, China

**Keywords:** Fano resonance, metal–dielectric–metal (MDM) waveguide, nanosensor, on-chip plasmonic structures, surface plasmon polaritons (SPPs)

## Abstract

A novel refractive index nanosensor with compound structures is proposed in this paper. It consists of three different kinds of resonators and two stubs which are side-coupled to a metal–dielectric–metal (MDM) waveguide. By utilizing numerical investigation with the finite element method (FEM), the simulation results show that the transmission spectrum of the nanosensor has as many as five sharp Fano resonance peaks. Due to their different resonance mechanisms, each resonance peak can be independently tuned by adjusting the corresponding parameters of the structure. In addition, the sensitivity of the nanosensor is found to be up to 1900 nm/RIU. For practical application, a legitimate combination of various different components, such as T-shaped, ring, and split-ring cavities, has been proposed which dramatically reduces the nanosensor dimensions without sacrificing performance. These design concepts pave the way for the construction of compact on-chip plasmonic structures, which can be widely applied to nanosensors, optical splitters, filters, optical switches, nonlinear photonic and slow-light devices.

## Introduction

Surface plasmon polariton (SPP) is a unique optical phenomenon which occurs in the coupling of electromagnetic waves with free electrons at the metal–dielectric interface [1]. It can overcome the classical diffraction limit of light. Based on this property, metal–dielectric–metal (MDM) waveguides have been designed and widely applied to manipulate light within sub-wavelength dimensions. Many plasmonic structures, such as high-sensitivity refractive index sensors [2], enhanced biochemical sensors [3], switches and filters [4], have been designed based on the concept of Fano resonance by utilizing a MDM waveguide [3,5–6]. Due to the interference of continuous (bright) modes and discrete (dark) modes, the Fano resonance exhibits a sharp asymmetric line shape characteristic [7], which has attracted more and more attention. The common design methods of these structures can be generally divided into three categories – First is that the input and output waveguides are direct coupled to both ends of the resonator [3,8–10], second is that the resonators are side-coupled to one waveguide between the input and output ports [11–15], and third is that the input waveguide, output waveguide and resonators are all coupled through a gap [2,16–17]. The common resonators are rectangular [6], ring [14], triangular [9], disk [18–19], hexagonal [20] and other special shapes. In recent years, many structures have been proposed to obtain the Fano resonance effect [20–21]. Obviously, a structure with only one resonant mode is hardly expected to have practical applications [20,22]. Therefore, structures that can excite multimode resonances are proposed [23]. It is known that increasing the number of transmission peaks can acquire more reliable results to improve the accuracy and the fault tolerance of the structure effectively. Independently adjusting the position of the resonance peaks can make the structure high suitability for different applications, and the compact size is always desirable in the design of on-chip plasmonic structures. However, multiple resonance peaks generally imply more complex structures resulting in difficulties in obtaining a highly independent tunability [24]. It is also a technical challenge to reduce the size of the structure while also guaranteeing high performance [15].

So far, there have been plenty of reports on dual/triple/quad Fano resonances for refractive index sensors on the basis of MDM waveguides. Normally, two or three different resonators are employed within a MDM waveguide environment, one of which effectively creates a continuous bright mode, and the other(s) discrete dark mode(s), or interference between different modes through the phase-coupling effect. The sharp response line of a Fano resonance is preferable to create an excellent plasmonic sensor with ultrahigh performance. However, such attempts to date lack the utilization of various resonators to generate multiple Fano peaks/dips at an arbitrary defined position for practical multi-sensing applications.

In this paper, we proposed a compact plasmonic nanosensor, which is composed of one MDM waveguide, two side-coupled stubs, and three gap-coupled resonators (a T-shaped, a ring and a split-ring, respectively). The transmission features of the structure are numerically simulated in the near-infrared spectrum at 1000 to 2000 nm by the finite element method (FEM). The simulation results show that the transmission spectrum has five Fano resonances with nearly 200 nm intervals between the different modes. Thus, each Fano resonance peak can be independently and precisely tuned by changing the parameters of the corresponding resonator. The characteristics of each resonance mode are further investigated in detail. By comparing these resonators to each other, it can be found that the resonator, when directly coupled to the MDM waveguide, has a higher transmission, the asymmetric T-shaped resonator can generate multiple resonant modes, the ring resonator can produce a sharper transmission peak, and the split-ring resonator has the minimum size with more adjustable parameters under the premise of guaranteeing high performance. Hence, our compound structure combines the advantages of the various resonators, such as the asymmetric T-shaped, ring and split-ring, to obtain multiple Fano resonance modes with highly compact dimensions and independent tuning of peak positions. Additionally, the research on the refractive index properties of the nanosensor shows that the maximum value of the sensitivity is 1900 nm/RIU and the figure of merit (FOM) is 1199. All these capabilities are considered to be excellent in comparison to similar, previously reported nanosensors. Thus, our structure has great potential for on-chip detection with high performance. Moreover, our study on the characteristics of the different types of resonators also provides a powerful theoretical guidance for all-optical integration systems and ultra-compact plasmonic devices.

## Modeling and Simulation

**Figure 1:**
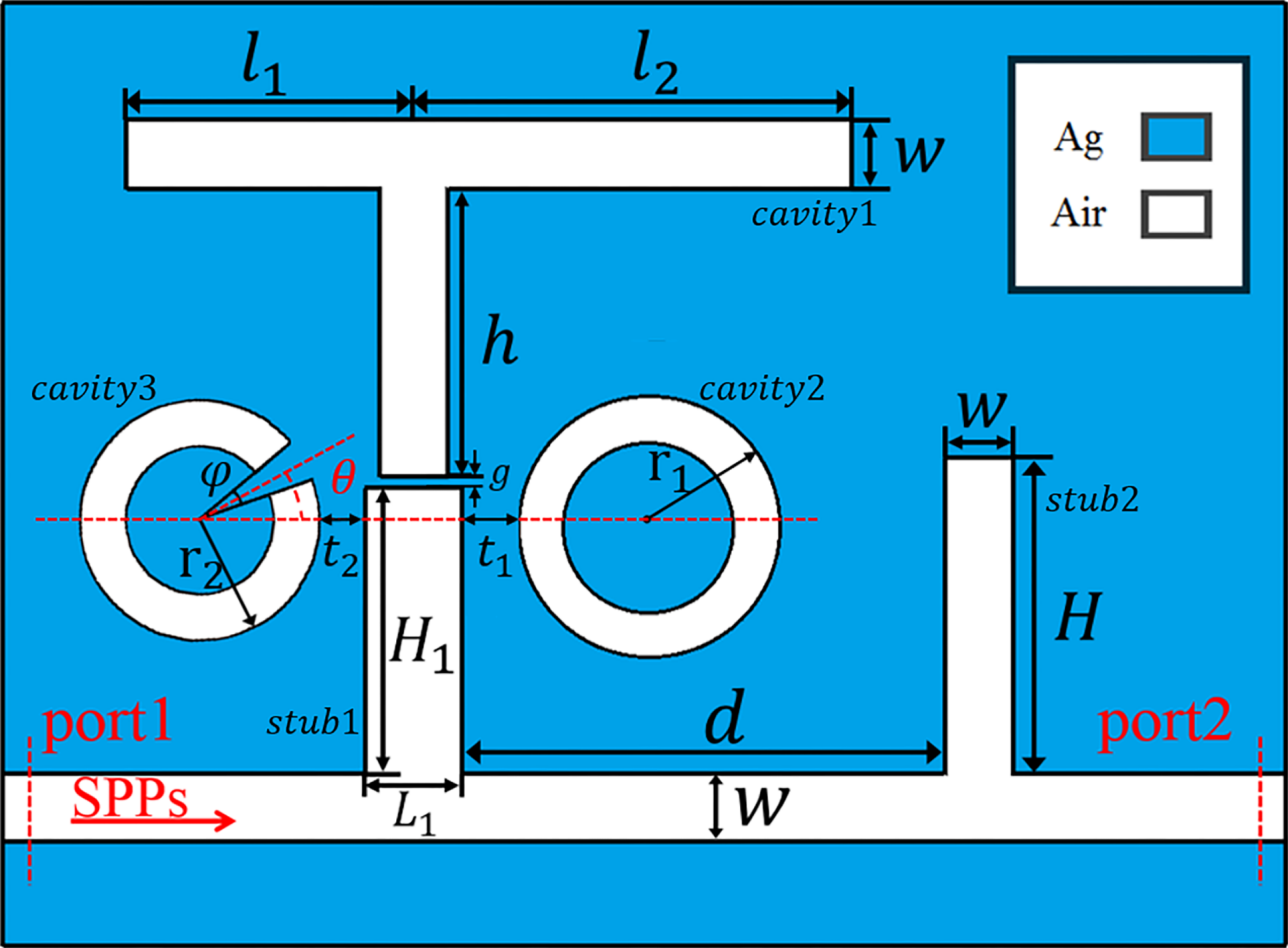
Schematic diagram of the plasmonic nanosensor. The geometric parameters are labelled on the structure for the following discussion.

Figure 1 shows a schematic diagram of the plasmonic nanosensor designed in this work. It is composed of three resonators (an asymmetric T-shaped, a ring and a split-ring resonator), which are gap-coupled to a bus waveguide with two stubs. For convenience, we named the T-shaped, ring, split-ring, left stub and right stub modules as cavity1, cavity2, cavity3, stub1 and stub2, respectively. The width of the bus waveguide, the three cavities and stub2 are fixed at *w* = 50 nm in this paper. *h* is the height of the vertical part of cavity1 and its horizontal length is divided into *l*_1_ and *l*_2_. *r*_1_ and *r*_2_ are the outer radius of cavity2 and cavity3, respectively. The opening angle of cavity3 is denoted by φ (here, φ = 20°). The angle between the center line of the opening and the horizontal axis (the red-dashed line in Figure 1) is marked as θ. *L*_1_ and *H*_1_ is the length and the height of stub1, respectively, while *H* is the height of stub2, and *d* is the distance between stub1 and stub2. The coupling distance between stub1 and cavity1 is represented by *g*. Similarly *t*_1_ and *t*_2_ are the coupling distance from cavity2 and cavity3 to stub1, respectively. The center of the cavity2 and cavity3 has the same distance to the bus waveguide, which is 215 nm. In the schematic diagram, the white and blue areas represent dielectric and metal materials, respectively. The dielectric in the waveguide and cavities is air, of which the relative permittivity is ε_d_ = 1. The metal is silver, with permittivity ε_m_ characterized by the Drude model covering the wavelength range of 1000 to 2000 nm [25] represented by

[1] $$\varepsilon_{\text{m}}\left(\omega \right)=\varepsilon_{\infty }-\omega_{\text{p}}/\left(\omega^{2}+i\omega \gamma_{\text{p}}\right)$$

where ε_∞_ = 3.7 is the electric constant at the infinite angular frequency, the bulk plasma frequency ω_p_ is 1.38 × 10^16^ rad/s, ω stands for the angle frequency of the incident wave, and the damping rate γ_p_ is 2.73 × 10^13^ rad/s, which characterizes the absorption loss. A TM-polarized plane wave is launched from port1 to excite the SPPs. Here *P*_in_ and *P*_out_ stand for input and output power flows of the input port1 and output port2, respectively. The transmittance is defined as *T* = *P*_out_/*P*_in_.

Since the width of the bus waveguide is much smaller than the wavelength of the incident light, only a single propagation mode TM_0_ can exist in the structure, of which the dispersion relation is determined by the following equation

[2] $$\begin{array}{l} \varepsilon_{\text{m}}\sqrt{\beta_{\text{spp}}^{2}-\varepsilon_{\text{d}}k_{0}^{2}}\tanh\left(\frac{w\sqrt{\beta_{\text{spp}}^{2}-\varepsilon_{\text{d}}k_{0}^{2}}}{2}\right)\\ +\varepsilon_{\text{d}}\sqrt{\beta_{\text{spp}}^{2}-\varepsilon_{\text{m}}k_{0}^{2}}=0 \end{array}$$

where β_spp_ = *k*_0_*n*_eff_ is the propagation constant of the SPPs in the waveguide, *n*_eff_ is the effective refractive index, and *k*_0_ = 2π/λ is free space wavenumber. Then the resonance wavelength of different modes for stub and resonators can be derived from the standing wave theory by the resonance condition as follows

[3] $$\lambda_{\text{stub}}=\frac{4n_{\text{eff}}L_{\text{eff}}}{\left(2m-1\right)-\varphi_{r}/\pi },\;m=1,2,3,...$$

and

[4] $$\lambda_{\text{res}}=\frac{2n_{\text{eff}}L_{\text{eff}}}{m-\varphi_{r}/\pi },\;m=1,2,3,...$$

where *L*_eff_ is the effective length of the cavity, and φ_r_ is the phase shift of SPPs reflected on the facets of the cavity.

The transmission characteristics of the plasmonic waveguide system can be analyzed by coupled mode theory (CMT). In this theory, the total field can be obtained by the superposition of various modes. When multiple modes are coupled in a narrow wavelength range, the phase difference of different modes cannot be ignored. Therefore, multimode interference coupled mode theory (MICMT) is proposed on the basis of CMT by adding a phase difference effect, and its equations are expressed as follows

[5] $$\frac{\text{d}a_{n}}{\text{d}t}=j\omega_{n}a_{n}-\left(\frac{1}{\tau_{n0}}+\frac{1}{\tau_{n1}}+\frac{1}{\tau_{n2}}\right)a_{n}+\kappa_{n1}S_{1+}+\kappa_{n2}S_{2+}$$

[6] $$S_{1-}=-S_{1+}+\sum_{n}\kappa_{n1}^{*}a_{n}e^{i\varphi_{n1}}$$

[7] $$S_{2-}=-S_{2+}+\sum_{n}\kappa_{n2}^{*}a_{n}e^{i\varphi_{n2}}$$

where *a_n_* and ω*_n_* are the field amplitude and resonant frequency of the *n*th mode, respectively. τ*_n_*_0_ is the decay time of internal loss of the *n*th mode in a resonant system. τ*_n_*_1_ and τ*_n_*_2_ are the decay time of the coupling between the resonant system and the left and right parts of the bus waveguide, respectively. κ*_n_*_1_ and κ*_n_*_2_ are the coefficients expressing the degree of the coupling between the resonant system and the waveguide. φ*_n_*_1_ and φ*_n_*_2_ are the complex amplitude phases of the *n*th resonant mode coupled to the waveguides. *S_i_*_±_ are the field amplitudes in each part of the waveguide (*i* = 1, 2) for outgoing (−) or incoming (+) from the resonator. In this paper, only port1 has TM wave incidence. The input and output port are symmetrical with the same *w* about the resonance system. Hence, *S*_2+_ = 0, τ*_n_*_1_ = τ*_n_*_2_ = τ*_n_*, and the transmittance *T* is satisfied by the following equation

[8] $$T={\left|t\right|}^{2}={\left|\frac{S_{2-}}{S_{1+}}\right|}^{2}={\left|\sum_{n}\frac{2e^{j\varphi_{n}}}{-j\left(\omega -\omega_{n}\right)\tau_{n}+2+\frac{\tau_{n}}{\tau_{n0}}}\right|}^{2}$$

where *t* is the transmission coefficient and φ*_n_* is the total coupling phase difference of the *n*th resonant mode.

## Results and Discussion

In this paper, the compound structure of our plasmonic nanosensor, as shown in Figure 1, is numerically investigated by the finite element method (FEM), which is also comparative to MICMT. The transmittance values are calculated according to the definition of Equation 8. It is well known that the excitation of Fano resonance requires the interaction of continuous (bright) modes and discrete (dark) modes. In our design, stub1 can form the bright mode, and each of the three resonators (cavity1, cavity2, cavity3) can generate the different dark modes. To further reveal the resonance properties, like assembling as building blocks, we performed a series of numerical simulations to discuss the mechanism of our design.

**Figure 2:**
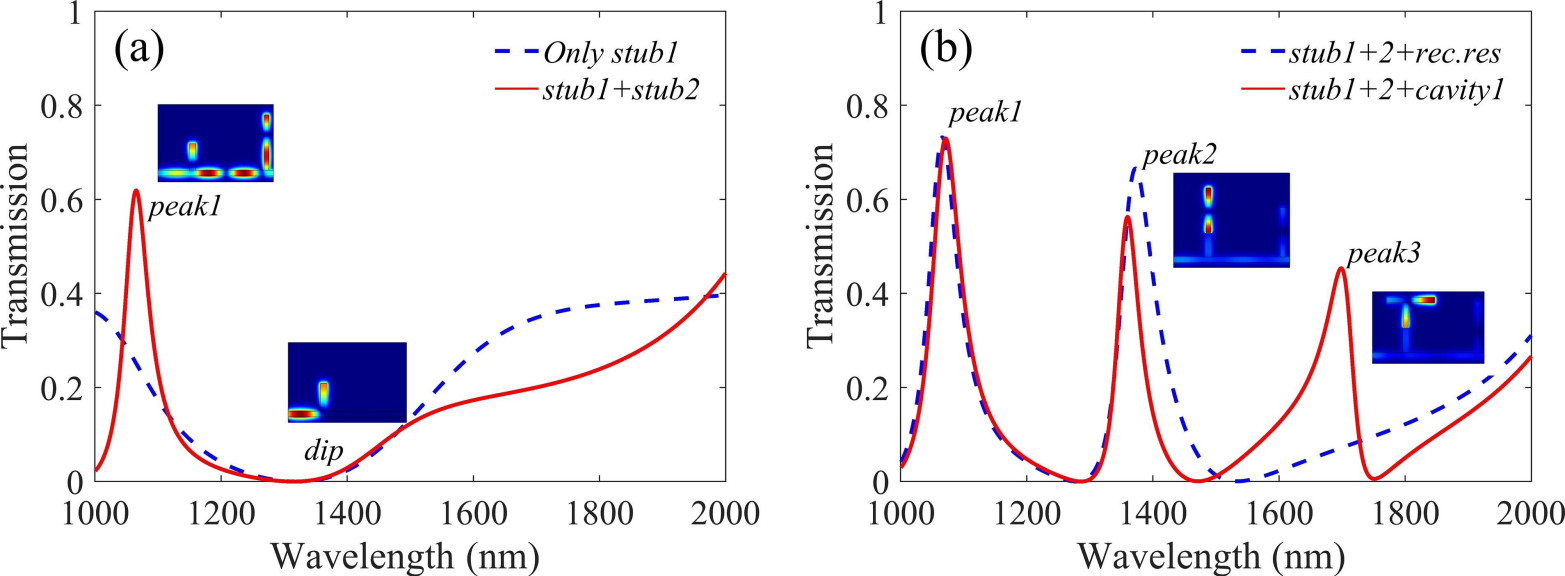
Transmission spectra of (a) only stub1 (blue dashed line), stub1 and stub2 (red solid line) side-inserted into the bus waveguide and (b) two stubs and a rectangular resonator (blue dashed line), two stubs and cavity1 (red solid line) in the structure.

Firstly, a simple layout consisting of the bus waveguide with stub1 was studied and the transmittance spectrum is depicted by the blue dashed line, as shown in Figure 2a. A single side-coupled cavity of different shape with stub1 can be compared to the performance of one Fano resonance peak as shown in Supporting Information File 1.

When stub2 is added in the beginning of a simple layout, one resonance peak is excited as shown by the red solid line in Figure 2a. The height of stub2 (*H*) and the distance between the two stubs (*d*) are set to 500 nm and 750 nm, respectively. Obviously, the blue dashed line shows a wide dip around 1320 nm, which provides the bright mode. While the dark mode is excited by stub1, stub2 and the middle part of the bus waveguide between these two stubs, interacting with each other to get the first peak at 1066 nm, which is denoted as peak1.

Based on the single mode system, a dual resonance mode system is constructed by side-coupling a rectangular resonator above stub1. The width and height of the rectangular resonator are 50 nm and 450 nm and the coupling distance *g* is 8 nm. The transmittance spectrum is shown in Figure 2b by the blue dashed line, where a second peak appeared at 1373 nm, labelled as peak2. In order to shrink the geometry and spare the level space, we replaced the rectangular resonator with an asymmetric T-shaped resonator named cavity1. The transmittance curve is plotted in Figure 2b with a red solid line. The size parameters of cavity1 are *l*_1_ = 210 nm, *l*_2_ = 320 nm and *h* = 240 nm, respectively. Because of the asymmetry of the T-shaped resonator (*l*_1_ ≠ *l*_2_), a third resonance peak emerges at 1699 nm, denoted as peak3. The effective length *L*_eff_ of cavity1 is *l*_1_ + *h* = 450 nm and *l*_2_ + *h* = 560 nm, corresponding to peak2 and peak3, respectively. The effective length *L*_eff_ in the rectangular resonator and cavity1 are the same, but in Figure 2b we can see peak2 has a slight shift in wavelength. This is because the *n*_eff_ of cavity1 is slightly larger than the rectangular resonator, therefore a slight blue shift occurs when cavity1 is substituted for the rectangular resonator. All these theoretical analyses are very consistent with the aforementioned Equations (Equation 2–4).

**Figure 3:**
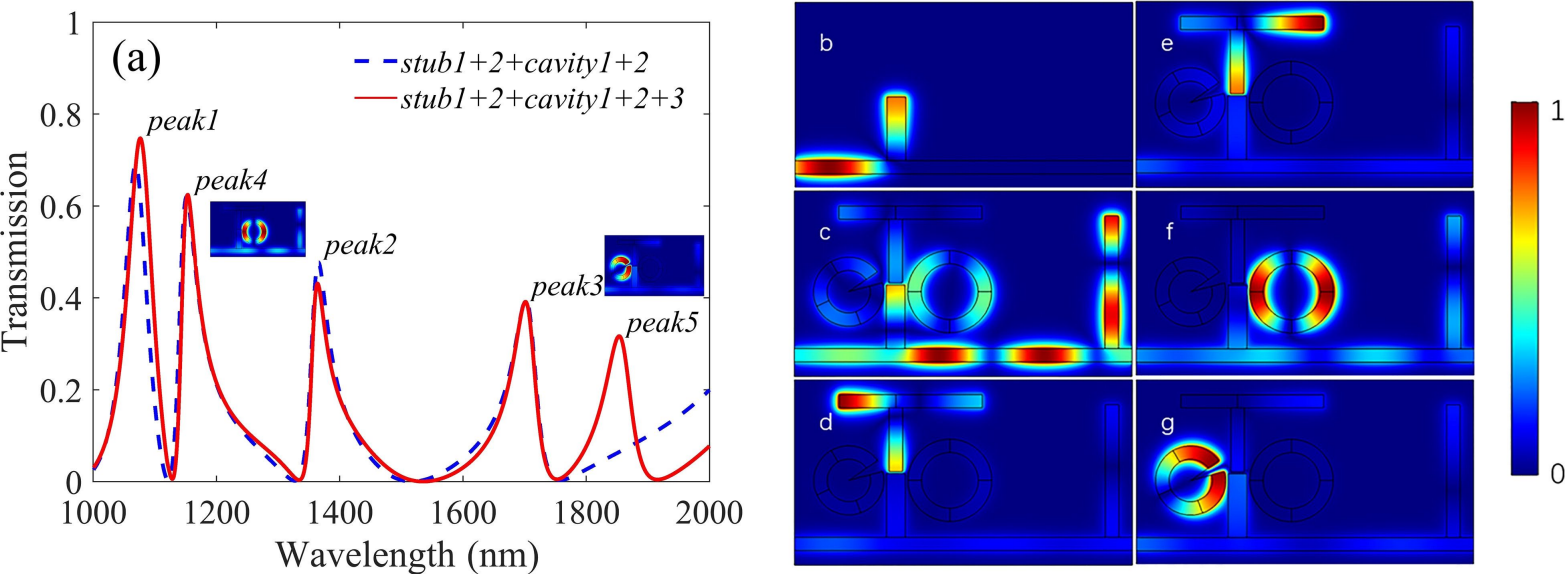
Transmission spectra of the structure with (red solid line) and without (blue dashed line) cavity3, and (b) the distribution of normalized magnetic field |*H*_z_| at 1320 nm with only stub1 inserted into the bus waveguide. The |*H*_z_| distribution of the total compound structure at the resonance wavelengths of (c) 1077 nm, (d) 1364 nm, (e) 1702 nm, (f) 1154 nm, and (g) 1854 nm is shown.

By coupling a ring resonator (cavity2) with a 155 nm outer radius and 10 nm coupling distance from the right side of stub1, a new resonance mode is induced near peak1, denoted as peak4. Also a new dip at 1122 nm between peak1 (1069 nm) and peak4 (1152 nm) appears, as shown in Figure 3a by the blue dashed line. This is attributed to the phase-coupling and the interference between the bright mode and various dark modes in a narrow wavelength range. Such dramatic changes from dip to peak can significantly improve the detection accuracy of the nanosensor.

Finally, a split-ring resonator (cavity3) is inserted into the left side of stub1. The relevant parameters of cavity3 are *r*_2_ = 130 nm, φ = 20°, θ = 25° and *t*_2_ = 9 nm. The corresponding transmission spectrum is shown in Figure 3a by the red solid line, where a new Fano resonance peak emerges at 1854 nm, denoted as peak5 due to the inserted cavity3. There is a slight deviation, which is mainly because of the interference of adjacent modes and the neglect of the wavelength dependence of φ_r_ [26–27].

Moreover, Figure 3b shows the |*H*_z_| field of the structure only composed of stub1 and a bus waveguide at λ = 1320 nm. According to Equation 2 and Equation 3, the incident and reflected waves in stub1 and the left part of the waveguide form constructive interference, while in the right part destructive interference occurs. Hence, the transmittance is almost zero, which is in good agreement with the situation of the dip inset in Figure 2a. Figure 3c–g corresponds to peaks 1–5, respectively. Figure 3c illustrates that the magnetic field energy of peak1 at 1077 nm is mainly concentrated on stub1, stub2 and the middle part of the bus waveguide between these two stubs. The |*H*_z_| of peak2 at 1364 nm is shown in Figure 3d, where almost all energy is limited to the left and bottom parts of cavity1. A similar situation for peaks 3, 4 and 5 are shown in Figure 3e, f and g, respectively. Obviously, each transmittance peak corresponds to a specific resonance element, which purposely provides a flexibility to the design of plasmonic devices with multiple Fano resonances.

**Figure 4:**
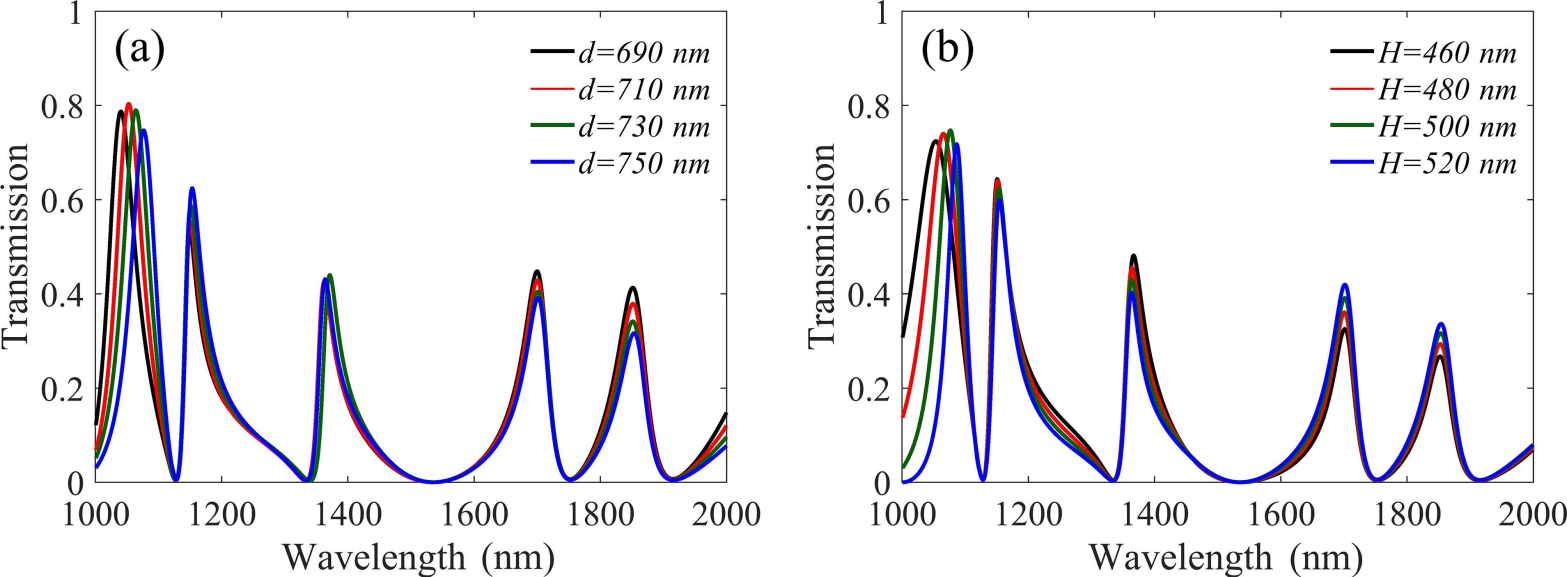
Dependence of the transmission on two parameters, (a) the distance *d* between two stubs and (b) the height *H* of stub2.

In the following part, we further investigated the parametric response of each resonance element and discussed the performance of the nanosensor. For the sake of a concise description, each time only one parameter has a variation, the other parameters are held constant. Figure 4a shows the transmission spectra of changing the distance *d* between two stubs. It was found that the resonance wavelength of peak1 will produce a significant red shift by increasing *d*, while the other four peaks remain almost unchanged. Figure 4b shows the resonance wavelength of peak1 also has a red shift by increasing the height *H* of stub2. It can be seen by comparing the Figure 4a and b that the change of the full width at half maxima (FWHM) of peak1 is more stable when the parameter *d* is adjusted. This is mainly because the change of *H* has a larger influence on the symmetry of the resonance system than the change of *d*. Moreover, the transmittance of peak1 is higher than the others, due to the direct coupling between the stubs and the waveguide. It is worth mentioning that the continuous bright mode is only excited by stub1, while the discrete dark mode can be excited by either of the three cavities. Thus, stub1 plays the important role to excite both the bright mode and the dark mode in this resonance system. A similar structural design could significantly reduce the device size in some specific situations.

**Figure 5:**
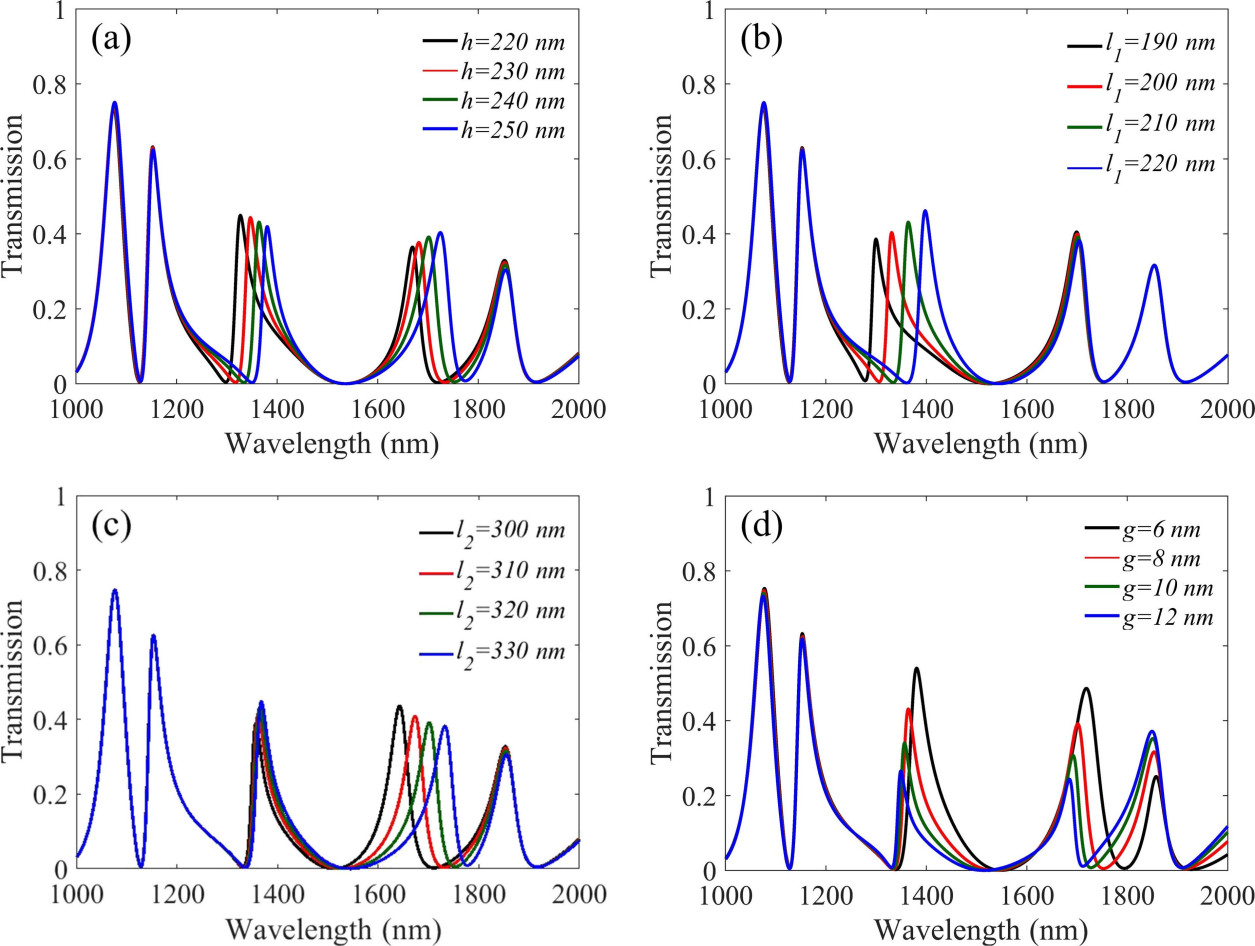
Transmission spectra of different parameters, (a) the height of cavity1 (*h*), (b) the left part of the horizontal length (*l*_1_), (c) the right part of the horizontal length (*l*_2_), and (d) the coupling distance of cavity1 (*g*).

Next, the influence of adjusting the parameters of cavity1 on the transmission spectra are discussed in detail. Firstly, the influence of the height of cavity1 *h* on the transmission spectrum is studied and the results are shown in Figure 5a. It can be seen that peak2 and peak3 have a significant red-shift when *h* is increased. The reason is that increasing *h* results in an increase in *L*_eff_ of these two resonance modes, thus increasing the resonance wavelength of peak2 and peak3. The results of changing *l*_1_ and *l*_2_ are shown in Figure 5b,c, respectively. Obviously, the length of *l*_1_ affects the position of peak2 and the length of *l*_2_ affects the position of peak3. All of these results are consistent with Equation 2 and Equation 4, because the changes correspond to the effective length *L*_eff_ of the resonator. Then, as shown in Figure 5d, the value of the coupling distance *g* has a great influence on the transmittance and the FWHM. When *g* is increased, the decay time τ*_n_* of the coupling between the resonant system and the waveguide will increase, and this can lead to the decrease in transmittance and FWHM, which is exactly in agreement with the theoretical analysis of Equation 5–8.

However, the good sensor performance requires high transmission and narrow FWHM. It is thus necessary to select the appropriate *g* to compromise these two parameters. As previously mentioned, peak2 is controlled by the left and bottom parts of cavity1, while the resonance of peak3 is produced by the right and bottom parts of cavity1. This kind of multiple use of the cavity can effectively decrease the size of the structure.

**Figure 6:**
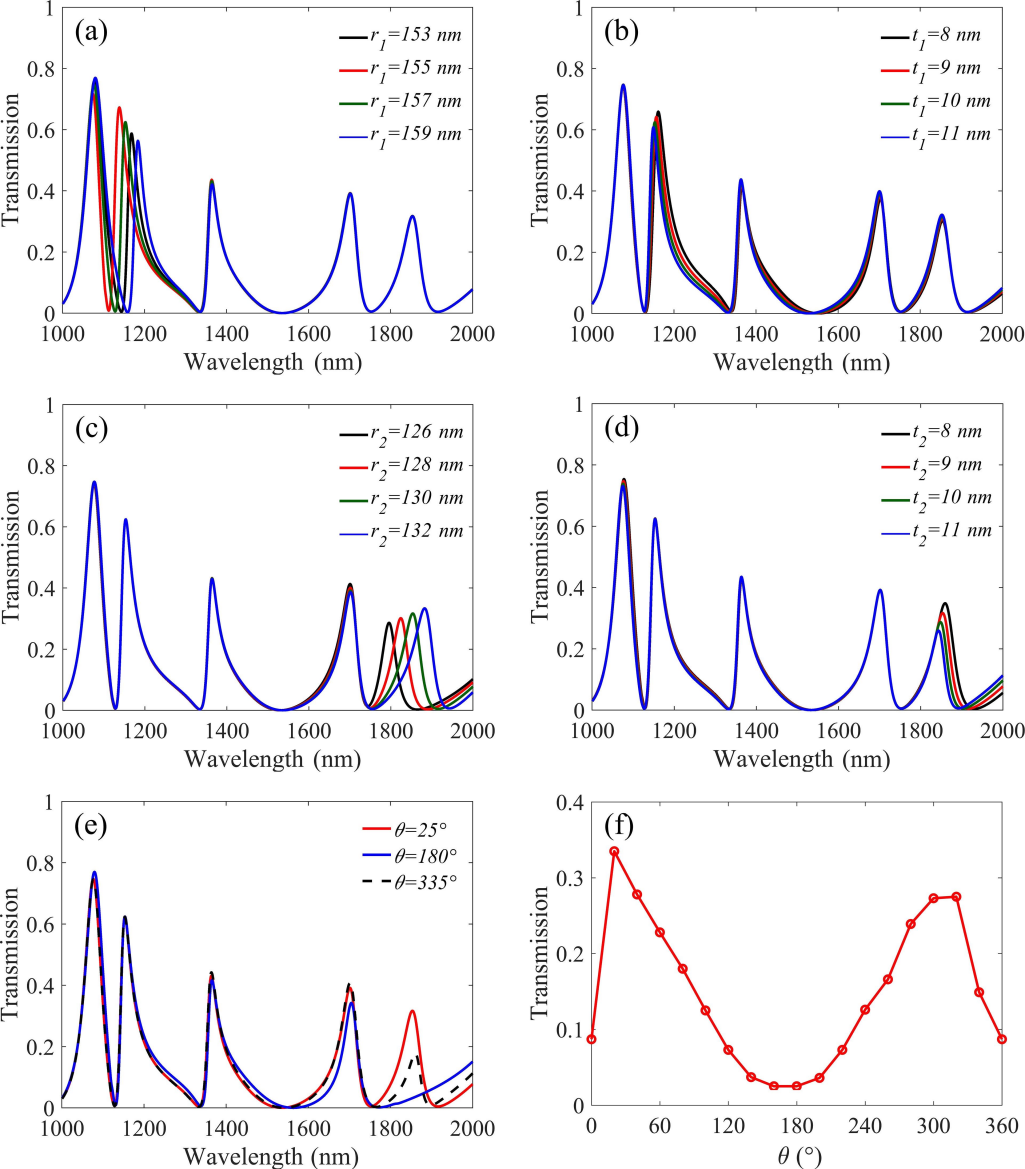
Transmission spectra of (a) the outer radius *r*_1_ of cavity2, (b) the coupling distance *t*_1_ of cavity2, (c) the outer radius *r*_2_ of cavity3 (d) the coupling distance *t*_2_ of cavity3 and (e) three specific values of θ. (f) The dependence of the transmission of peak5 on θ.

Subsequently, we investigated the features of cavity2 and cavity3 on sensing performance. Figure 6a shows the relation between *r*_1_ and the transmission spectra. When the outer radius *r*_1_ of cavity2 is increased, a significant red shift appears in peak4, while the other peaks are stable. A similar situation occurs for the outer radius *r*_2_ of cavity3 and peak5, as shown in Figure 6c. In Figure 6b, d, we can see that the coupling distance *t*_1_ and *t*_2_ simultaneously affect the FWHM, the resonance wavelength position and the transmittance of peak4 and peak5. This is quite similar to the case of the coupling distance *g* of cavity1 discussed above. Therefore, *t*_1_ and *t*_2_ are also not suitable parameters for independent tuning. The tilt angle θ of cavity3 also affects the performance of the nanosensor. Figure 6e shows the spectra under three special values of θ. Obviously the red and blue lines have five peaks whilst the black dashed line has only four, and peak5 will disappear when θ is 180°.

**Figure 7:**
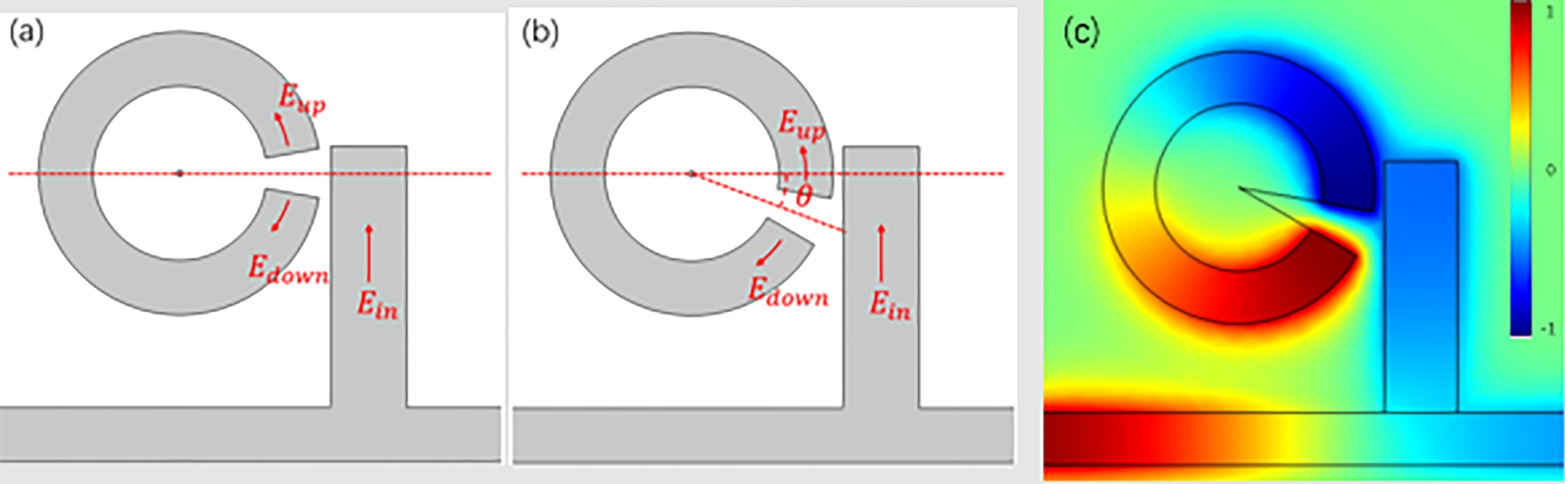
Schematic diagram of cavity3 with different tilt angles θ, (a) θ = 0°, (b) θ = −20°. (c) The distribution of the magnetic field *H*_z_ at θ = −20°.

In order to further reveal this interesting phenomenon, we simulate the dependence of the transmission on θ and the result is shown in Figure 6f. The peak values of the transmission appear when θ = 20° and θ = 340° (i.e., −20°), while the dip values correspond to θ = 0° and θ = 180°. These maximum or minimum values are not rigorous symmetry, which is attributed to the limited height of stub1 and its asymmetrical coupling area with the split-ring, as shown in Figure 7.

Afterwards, for the convenience of the following analysis of cavity3, the electric field coupled into cavity3 is divided into up and down parts named *E*_up_ and *E*_down_, as marked with red arrows along the split-ring in Figure 7. When θ = 0°, as shown in Figure 7a, *E*_up_ and *E*_down_ are the mirror counterparts, which produced a typical destructive interference, resulting in low transmission. A similar phenomenon happens when θ = 180°. When θ is closer to 20°, the electric field is coupled to one end of cavity3, *E*_up_ almost disappears thus *E*_down_ dominates the whole coupling process, therefore, a high transmission is generated, which is consistent with the Equation 4. When θ is around 340° (i.e., −20°), as shown in Figure 7b, one end of cavity3 is much closer to stub1, thus the corresponding coupled field *E*_up_ and *E*_down_ of the other end is as shown in Figure 7c. This will lead to a slight destructive interference. Compared to the widely used ring resonator, the split-ring takes up only almost a quarter of the area to achieve analogous performance. In addition, the split-ring can be considered as a rectangle rolled up, which can save space compared to a conventional rectangular design. Besides, the tilt angle θ can be used as a free tuning switch for the structure design.

According to the analysis above, we can conclude that each resonance mode of our structure has an excellent independence tuning performance.

It is worth mentioning that the sensitivity, defined as *S* = Δλ/Δ*n*, and figure of merit (FOM) are important parameters for sensors. Here Δ*n* represents the variation of the refractive index in the surrounding environment and Δλ is the wavelength shift caused by the change of refractive index. The FOM is defined as FOM = Δ*T*/*T*Δ*n*, where *T* is the transmission of the structure and Δ*T*/Δ*n* denotes the transmission change at the fixed wavelength induced by a refractive index change.

**Figure 8:**
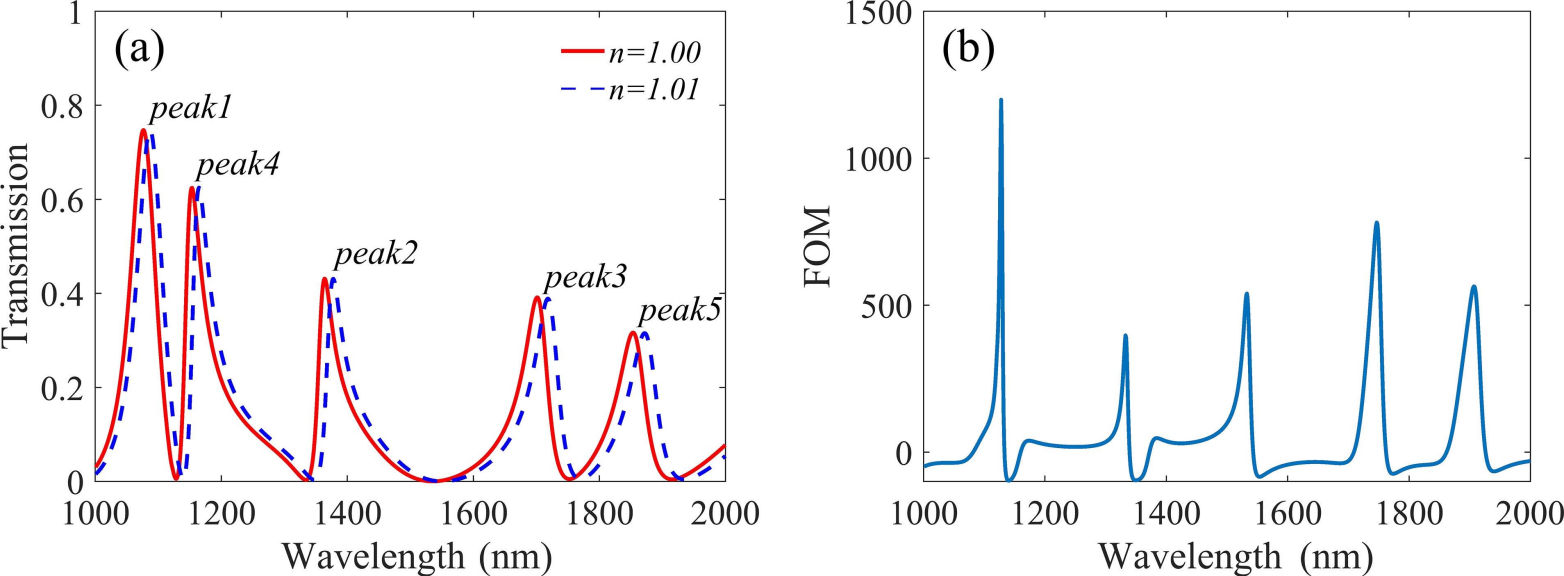
(a) Transmission spectra for different refractive index values, and (b) the FOM curve of the structure.

Figure 8a shows the transmission spectra when the refractive index of the surrounding environment changes. We can see that a small increase in the refractive index will lead to a significant red shift in the whole spectrum. Then we calculate the sensitivity of each resonance peak, and the results are 1000 nm/RUI, 1400 nm/RIU, 1600 nm/RIU, 1100 nm/RIU and 1900 nm/RIU corresponding to peak1, peak2, peak3, peak4 and peak5, respectively. The step difference of the sensitivity could allow for a wider variety of applications. The FOM curve is depicted in Figure 8b. It can be seen that there is a local maximum value of FOM at each dip in the transmission spectrum. The maximum value of the FOM is about 1199 at 1128 nm.

Next, we selected typical structures published in recent years for comparison [28–29], and the results are shown in Table 1 below. Obviously, the structure we propose has the most resonance peaks, covering a wavelength range of about 1000–2000 nm in the near-infrared region with relatively high sensitivity and FOM. Although, the FOM value is less than the outstanding values in previous reports, it is worth noting that our structure has the best independent tunability among the structures.

**Table 1:** Comparison of the proposed plasmonic sensor and other published similar solutions.

Ref.	Number of modes	Sensitivity (nm/RIU)	FOM	Independently tunable?

[3] (2014)	2	550/600	860/660	no
[8] (2016)	3	600/500/500	3803/816/2947	partially
[9] (2018)	4	412/520/866/986	32870/16410/324600/5003	no
[13] (2018)	3	1000/1400/1900	7500/8600/7000	partially
[15] (2018)	4	200/600/600/2000	3000/500/1500/200	partially
[16] (2017)	2	677/718	1795/4354	no
[20] (2018)	1	610	250	yes
[24] (2018)	1	880	964	yes
[28] (2019)	2	664/1792	1214/3804	no
[29] (2019)	2	624/924	21/41	no
this work	5	1000/1100/1400/1600/1900	1199/398/540/781/564	yes

**Figure 9:**
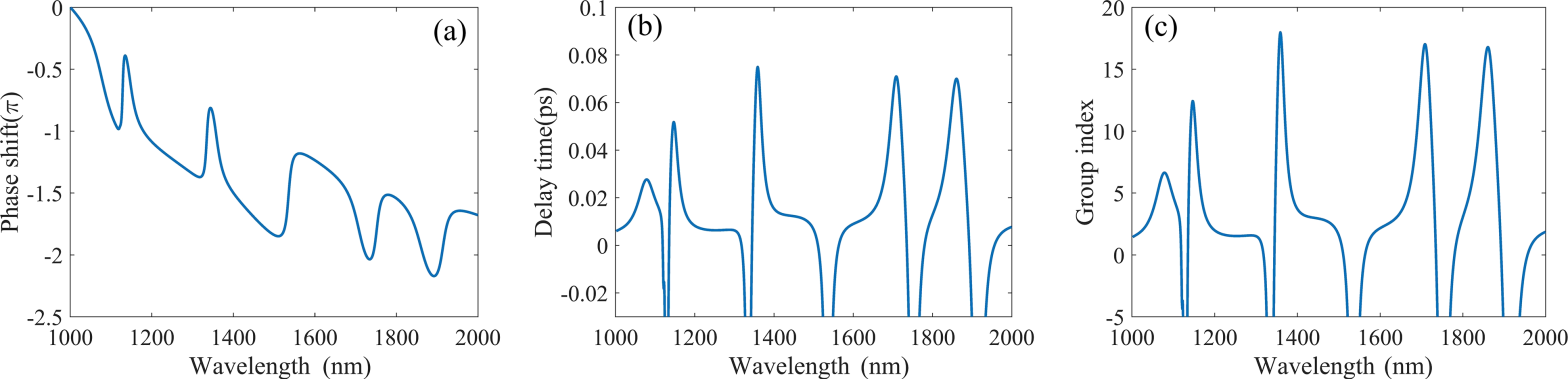
Dependence of (a) phase shift, (b) delay time, (c) group index on the wavelength.

It is worth noting that this structure not only displays excellent performance in refractive index sensing, but also shows high potential as a slow light device. In the design of slow light devices, the group refractive index is an important parameter to evaluate the performance of the structure. The group refractive index of the structure can be calculated by using the phase shift property. Figure 9a shows the phase shift diagram of the structure. From the figure we can see that there is a noteworthy phase shift at each resonance peak. The phase shift can be converted into delay time by τ(λ) = dϕ(ω)/dω. Then the calculated delay time is shown in Figure 9b. The maximum delay time reaches to 0.075 ps, which leads to the slow light effect.

Then the group refractive index and group velocity were calculated by the formula

[9] $$n_{\text{g}}=\frac{c}{v_{\text{g}}}=\frac{c}{D}\tau_{\text{g}}=\frac{c}{D}\frac{\text{d}\phi \left(\omega \right)}{\text{d}\omega }$$

where *n*_g_ is the group refractive index, *v*_g_ is the group velocity, *c* represents the speed of light in free space and *D* is the distance between the input port and the output port. Figure 9c shows the dependence between the group refractive index and the incident wavelength. It can be seen that the group refractive index is up to 18 at 1359 nm which is much higher than similar devices and this result is due to its compact size. The dip in the transmission spectra can also produce a fast light effect due to the presence of anomalous group velocity dispersion. Based on Figure 8a and Figure 9c, it can be concluded that the group refractive index obtained at peak2 is 16, and its transmission can also reach 0.43, which confirms excellent performance for a slow light device. Thus, the structure proposed in this paper can also provide a theoretical basis for the slow light structure design in the field of nano-integrated photonic devices.

## Conclusion

In summary, we report a novel nanosensor that is composed of two stubs and three resonators coupled with a MDM waveguide. The results obtained by FEM show that the structure produces five sharp Fano resonances, where each of them can easily tuned independently by changing the specific parameters. After a series of simulation tests, we learned that the coupling distance between the different cavities needs to be optimized and the structural size parameters are more conducive to free tuning of the position of the resonance modes. The multiple cavities, the use of asymmetric structures and a reasonable combination of different resonators can work together to produce a design that dramatically reduce the structure dimensions without sacrificing performance. Furthermore, the plasmonic nanosensor has a maximum sensitivity of up to 1900 nm/RIU. Compared with similar devices, the more compact size and the ease of tunability are the most outstanding advantages of the structure. The analysis of asymmetric structures in this paper will provide a powerful theoretical guidance for future plasmonic device design. In addition, our structure may have important potential applications for compact on-chip plasmonic nanosensors, slow-light devices, spectral splitters, switches, and nonlinear photonic devices.

## Supporting Information

File 1 Additional calculations of the individual components.
