# Supplementary material for: Plasmonic nanosensor based on multiple independently tunable Fano resonances
Source: Beilstein J Nanotechnol. 2019 Dec 17;10:2527–37. doi: 10.3762/bjnano.10.243 (PMC6941414; doi:10.3762/bjnano.10.243)
Supplement: File 1 — Additional calculations of the individual components. [file Beilstein_J_Nanotechnol-10-2527-s001.pdf]

BEILSTEIN JOURNAL OF NANOTECHNOLOGY

Supporting Information

for

Plasmonic nanosensor based on multiple independently tunable Fano resonances

Lin Cheng, Zelong Wang, Xiaodong He and Pengfei Cao

Beilstein J. Nanotechnol. **2019,** *10,* 2527–2537. doi:10.3762/bjnano.10.243

Additional calculations of the individual components

License and Terms: This is a supporting information file under the terms of the Creative Commons Attribution License (https://creativecommons.org/licenses/by/4.0). Please note that the reuse, redistribution and reproduction in particular requires that the authors and source are credited.
The license is subject to the *Beilstein Journal of Nanotechnology* terms and conditions: (https://www.beilstein-journals.org/bjnano)

This part discusses the various types of single resonators, such as the rectangular, disk, ring or split-ring, side-coupled with stub1 and MDM bus waveguide before being combined into a complex structure. In order to supply more references on different components, we compare the performance of single common cavity within the same condition. We set the same parameters of the material, the bus waveguide and stub1, the default width is 50 nm and the coupling gap is 10 nm in distance for the following simulations.

Given a defined resonant wavelength $\lambda_{\mathrm{res}} = 1550\ \mathrm{nm}$, there are six typical modes on resonance with the *z* component of the magnetic field (H_z) distribution depicted in Figure S1. The geometry size has been calculated as: outer radius of ring is 202 nm, radius of disk is 421 nm, height of rectangle1 is 1081 nm as to the second order mode, the height of rectangle2 is 516 nm corresponding to the first order mode, the split-ring1 with opening angle 30° and outer radius 213 nm corresponding to the second order mode, the split-ring2 with opening angle 30° and outer radius 115 nm corresponding to the first order mode.

The performance characteristics of these resonators are compared in detail, as shown in Figure S2. Through the comparison diagram, for the same type of cavity, the 1st-order mode has a smaller cavity volume and higher transmission than its 2nd-order mode counterpart. It is quite interesting that the higher order mode has a smaller value of the FWHM, which is worthy of optimization for a further discussion. For different type of cavity, rectangular and

split-ring resonator have closer spectral lines, disk resonator has the smallest FWHM with the largest volume in dimension.

As the table inset in Figure S2, the sensitivity of these six modes are the same of 1500 nm/RIU, owing to the same environment of each cavity with the variety of size. And the FOM* is the maximum value of FOM. The disk has the better performance with the least FWHM and largest FOM*, as well as the largest volume in dimension. When the dimension limited, we did not choose the disk cavity in this manuscript because the volume of disk cavity is too large to be integrated into our multi-mode structure. Hence in the future, if ignoring the dimension limited, the disk cavity can be fully utilized as the cell to design new type of nanosensor.

In summary, according to the specific needs, these cavities can be chosen as a building block cell to form complex structures with exciting Fano resonances at any defined wavelength in visible and near-infrared region.

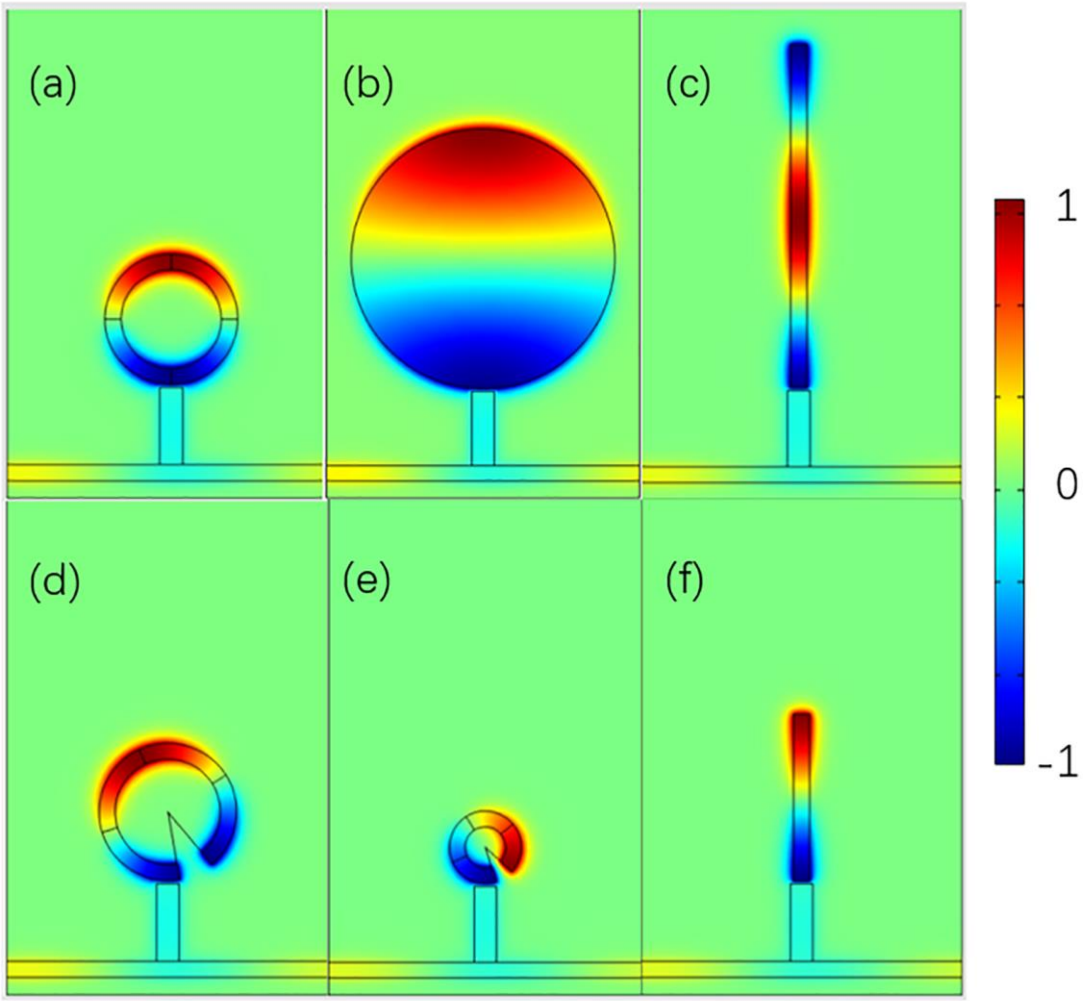

Figure S1: At resonance wavelength of 1550 nm, the *z* component of the magnetic field (Hz) distribution on (a) ring (outer radius is 202 nm), (b) disk (radius is 421 nm), (c) rectangle1 (height is 1081 nm correspond to the second order mode) (d) split ring1 (opening angle is 30°, and outer radius is 213 nm corresponding to the second order mode), (e) split ring2 (opening angle is 30°, and outer radius is 115 nm corresponding to the first order mode), (f) rectangle2 (height is 516 nm correspond to the first order mode), respectively.

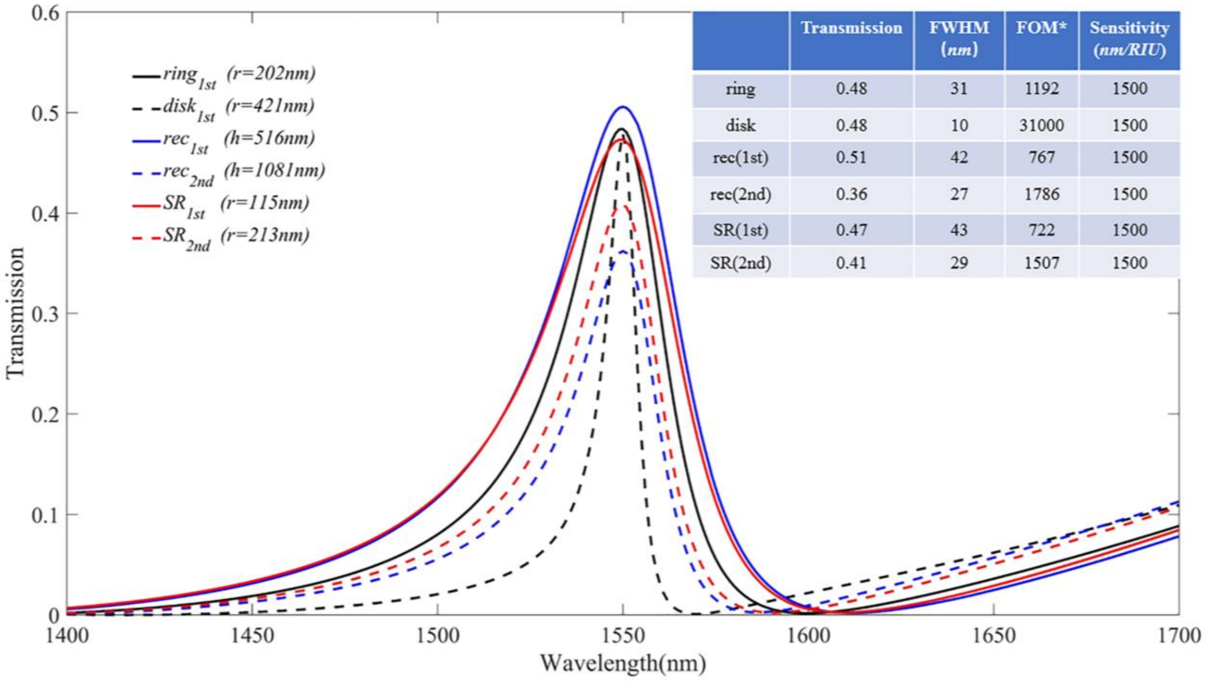

	Transmission	FWHM (*nm*)	FOM*	Sensitivity (*nm/RIU*)
ring	0.48	31	1192	1500
disk	0.48	10	31000	1500
rec(1st)	0.51	42	767	1500
rec(2nd)	0.36	27	1786	1500
SR(1st)	0.47	43	722	1500
SR(2nd)	0.41	29	1507	1500

Figure S2: The transmission spectra of six resonance modes. The peak value of transmission, FWHM, and maximum value of FOM (i.e., FOM*) are listed inset.
